# New Drugs, Appliances, and Things Medical

**Published:** 1891-09-19

**Authors:** 


### NEW DRUGS, APPLIANCES, AND THINGS MEDICAL.

[All preparations, appliances, novelties, etc., of which a notice is desired, should be sent for The Editor, to care of The Manager, 140, Strand, London, W.C.]

### BRITISH MEDICAL ASSOCIATION.

ANNUAL EXHIBITION.

Continuing our notes from last week we next come to MESSRS. BURROUGHS AND WELLCOMES, Snow Hill Buildings, E.C. This year they seemed to have, if possible, a larger and more varied exhibit than ever. An endless series of "tabloids" of every conceivable drug. Atomisers for the use of liquid ointments to the naso-pharynx. Cachets composed of pure rice starch for endorsing nauseous medicines, a great improvement on wafer papers. Hypodermic pocket cases. Inhalers of all sorts. Kepler's well-known preparations of malt. Malt with cod-liver oil. Lanoline and its preparations. Pareoline, an odourless, colourless parafin oil, which does not become rancid, and easily dissolves a large number of medicinal substances. It is peculiarly suitable for the atomisers just mentioned. A very excellent glycerine suppository is manufactured by this firm. It consists of a cone of cocoa butter containing the glycerine unmixed. We have found these decidedly useful in practice. A long list of digestive ferments and their products with the numerous ingenious inventions of Dr. Ward Cousins, such as ear-drums, rectal injectors added variety to the stall.

CADBURY BROTHERS, Bournville, near Birmingham, exhibited their well-known cocoa preparations, of which, it may be said, none better exist. Their announcement in the catalogue is significant "Cocoa. Absolutely pure."

WARRINGTON CHEMICAL COMPANY (Alfred H. Mason), 46, Jewin Street, E.C., showed their choloroform. We noted some time back this as being, according to our tests, a remarkably pure drug. It is prepared by the Ketone process, and is not thus likely to contain the impurities sometimes met with in alcohol-made chloroform. The cost is about one-third of the alcohol-derived drug. Personally we have used it repeatedly with excellent results.

SEABURY AND JOHNSON, 46, Jewin-street, showed a wonderful variety of surgical and medicinal plasters, dressings, and antiseptic dressings. We commented on these in our columns some time back very appreciatively. In addition to ordinary plasters Messrs. Seabury and Johnson have an excellent ready-for-use linseed poultice, which only requires moistening in hot water before applying, and as it is capable of being cut up into the desired size is very economical as well as being light, cleanly, and portable. The firm also showed their various preparations of the antiseptic hydronaphthol. Besides these there were sulphur candles for disinfection, and a pretty exhibition of medicated toilet soaps.

AYLESBURY DAIRY CO. (LIMITED), 31, St. Petersburg Place, Bayswater, W.—This well-known milk supply company had a good show of their various milk-derived preparations, as Koumiss, both "still" and "sparkling," which for many invalids is the only form in which they can take milk. "Humanised Milk." This is cow's milk so manipulated as to have the same percentage composition as human milk. It It has the advantage of keeping, when unopened, several weeks. There are also a "Special Milk Food for Infants," and Peptonised Milk, whey prepared without acid, and a general every-day drink of the Koumiss variety, called "Sparkling Bland."

### "FRAME FOOD" COMPANY.

In our recent notice of the above we referred to the very palatable and nutritious Frame Food Jelly and Drink Syrup, as "the extract combined with a fruit jelly." We learn from the manager of the company that "except having a little lemon acid to flavour them, they contain no fruit whatever, and consist only of extract and sugar." We regret that a slip of the pen has occurred, but hope that the above correction will rectify it. We have pleasure in again commending these foods to the consideration of those who require nutrients in a highly concentrated form.

